# Anterior Hyperfunction Syndrome: Literature Review and Conceptual Model

**DOI:** 10.3390/clinpract14040128

**Published:** 2024-08-18

**Authors:** Benjamin Aranda-Herrera, Tania Rubi Agudo-de la Cruz, Carlos Alberto Jurado, Rene Garcia-Contreras

**Affiliations:** 1 Interdisciplinary Research Laboratory, Nanostructures, and Biomaterials Area, National School of Higher Studies (ENES) Leon, National Autonomous University of Mexico (UNAM), Leon 37684, Mexico; benja.aherrera@gmail.com; 2 Private Clinical Practice, A&A Advanced Dentistry, Puebla 72340, Mexico; ponyta_nia@hotmail.com; 3 Operative Dentistry Division, Department of General Dentistry, College of Dentistry, University of Tennessee Health Science Center, Memphis, TN 38163, USA; cjurado@uthsc.edu

**Keywords:** Kelly syndrome, anterior hyperfunction syndrome, combined syndrome, dentistry

## Abstract

Combined Kelly syndrome, also known as anterior hyperfunction syndrome, is a complex pathological condition of the stomatognathic system, originally established by five characteristics but primarily triggered by edentulism, specifically, the absence of the upper and posterior mandibular teeth. This condition is characterized by a series of clinical features, such as bone loss, tuberosity growth, enamel wear, periodontal damage, muscle fatigue, pain, and temporomandibular joint issues. However, these features are not unique and rather reflect an oral hyperfunction state. There is a lack of consensus on the best way to assess and diagnose this condition, which is proposed to be understood as an “oral hyperfunction state” rather than a syndrome. This study aims to conduct a literature review to analyze the available information on anterior hyperfunction syndrome in dentistry, with the goal of proposing a conceptual model of the etiological risk factors that contribute to early diagnosis and the prevention of complications. This approach has important clinical implications, as it would allow for the early identification and management of risk factors, thus improving the quality of life of patients and preventing malpractice that could compromise their oral health.

## 1. Introduction

**Figure 1:**
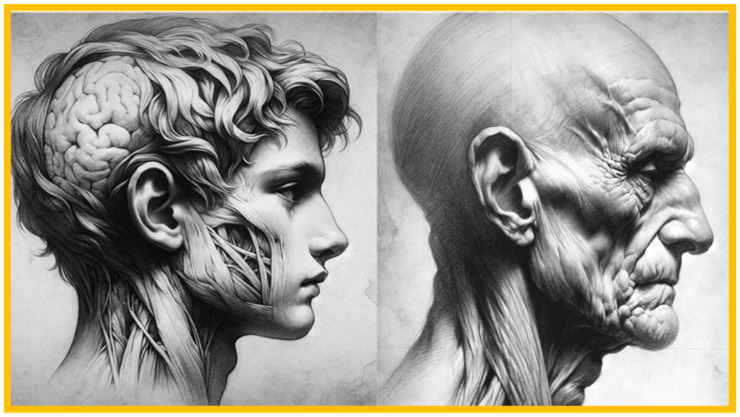
Physiognomic aspects of anterior hyperfunction syndrome (AHS). The left image depicts a young patient in apparent good health, exhibiting normal muscular characteristics and profile metrics. In contrast, the right figure represents a patient with AHS. Here, a decrease in facial height, the loss of muscular tone, the downturn of the corners of the mouth, a seemingly flattened face due to the loss of canines, and sunken cheeks indicative of an aged appearance are observed. These physiognomic features highlight the manifestations associated with the syndrome. Source: Dall-E2, OpenAI 2024.

Combination syndrome (CS) or Kelly’s syndrome (KS), also called anterior hyperfunction syndrome (AHS), is defined as a complex pathological state of the stomatognathic system with changes in the hard and soft tissue of the mouth that lead to occlusion caused by the remaining antero-inferior mandibular teeth, and the early maxillary anterior loss of bone is identified as the pivotal factor influencing other associated changes (Figure 1) [1].

**Figure 2:**
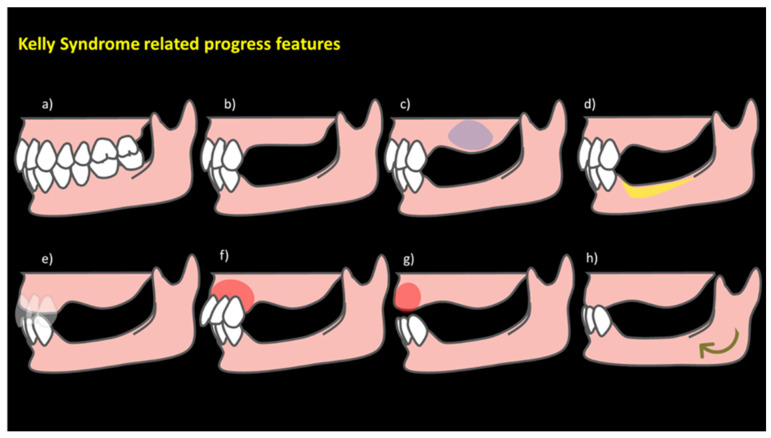
Visual progression of anterior hyperfunction syndrome (AHS) features. Illustrates the traditional features, providing a visual representation of the associated oral manifestations. (**a**) represents a healthy jaw, establishing the baseline; (**b**) indicates reduced vertical occlusion, suggesting the closer proximity of the dental arches; (**c**) depicts abnormal maxillary tuberosity enlargement due to maxillary sinus pneumatization; (**d**) highlights the significant thinning of the mandibular edentulous ridge; (**e**) shows the extrusion of the lower anterior teeth; (**f**) shows anterior maxillary crest bone loss; (**g**) reveals papillary hyperplasia on the hard palate; (**h**) displays mandible forward repositioning (indicated by the green arrow). These stages visually guide the characteristic dental and skeletal changes in Kelly syndrome, offering insights into the progression of associated oral features. Source: the authors.

AHS is described to be composed of five clinical changes: (1) bone loss of the anterior part of the maxillary crest; (2) excessive growth of the tuberosities; (3) papillary hyperplasia in the hard palate; (4) extrusion of the lower anterior teeth; and (5) excessive resorption of the mandibular edentulous ridges. However, Saunders et al. observed six other characteristics related to AHS, among which the following are described: (6) loss of vertical dimension in occlusion; (7) discrepancy in the occlusal plane; (8) anterior repositioning of the mandible; (9) poor adaptation of prostheses; (10) epulis fissuratum; and (11) periodontal changes (Figure 2) [2]. AHS is presumed to denote excessive occlusal force in the anterior region, which may result in bone loss in the front part of the maxillary ridge and simultaneously in the mandibular edentulous ridges beneath the removable partial denture (RPD), based on a phenomenon outlined by The Glossary of Prosthodontic Terms: Ninth Edition (GPT-9). In cases of combination syndrome, an overly strong bite from the remaining mandibular front teeth is presumed, regardless of the existence of the maxillary front teeth. Such circumstances commonly occur in individuals who have lost support in the back of their bite but still have their front mandibular teeth [3,4,5]. 

The complete denture experiences a loss of support and stability, initiating a rocking motion with upward movement (intrusion) in the anterior region and downward movement (extrusion) in the posterior region, with the fulcrum point or axis of movement positioned at the level of the canine or first premolar. The rocking motion generates negative pressure, akin to a suction chamber, behind the fulcrum line, causing the enlargement of the tuberosities and papillary hyperplasia in the upper complete denture’s posterior seal. Furthermore, the absence of posterior teeth in the maxilla reduces proprioception, leading to the pneumatization of the maxillary sinuses, resulting in only basal bone remaining [6].

**Figure 3:**
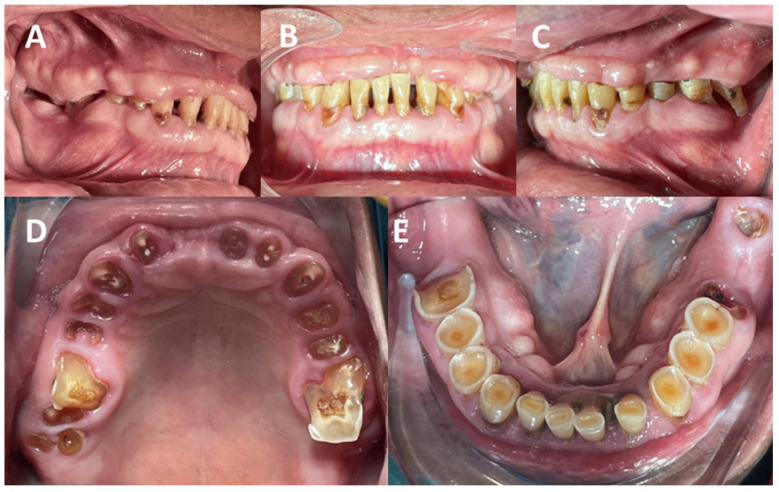
Clinical anterior hyperfunction syndrome (AHS) signs associated with temporomandibular disorders (TMD) and occlusal pathologies. The significant damage to the hard and soft tissue is shown, compromising not only aesthetics and function but also the patient’s state of homeostasis. It is necessary to perform an individual diagnosis of each tooth but also in an integral manner since the evidence shows a multifactorial etiology. (**A**–**C**) show the vertical growth of the tuberosity, as well as the state of anterior hyperfunction, in addition to the extrusion of the molars. There are clinical signs associated with exostosis. There is a reduction in the vertical dimension, which also causes the anterosuperior rotation of the mandible. Carious lesions secondary to abfractions are also evident. (**B**) shows the increased anterior Spee’s curve, causing trauma in the anterior region of the maxilla, as well as bone loss. (**D**,**E**) show severe attrition and erosion with pulpal exposure, in addition to dental fractures. Source: the authors.

AHS is typically caused by the overuse or hyperfunction of the masticatory system, which can stem from various factors, such as bruxism (the involuntary grinding or clenching of the teeth, often during sleep), malocclusion (poor alignment of the teeth and jaws, leading to the uneven distribution of occlusal forces), habitual chewing (like excessive gum chewing or favoring one side of the mouth), and psychological factors like stress and anxiety, which can lead to increased muscle activity and teeth grinding, which is also exacerbated by the anterior force component (AFC), which has been exposed as part of the natural aging process) [7]. Patients with AHS often present excessive wear of the tooth enamel, leading to sensitivity and a higher risk of cavities; damage to the periodontal ligaments, resulting in gum recession and tooth mobility; muscle fatigue and pain, which can extend to myalgia and headaches; and issues with the temporomandibular joint (TMJ), such as pain, clicking, or locking (Figure 3) [8].

Consequently, the treatment plans reported in the literature encompass a wide range of approaches, depending on the severity and specific manifestations of AHS. These approaches can include pre-prosthetic surgery to address underlying anatomical issues, the combination of fixed partial dentures (FPD) and RPD utilizing both intra- and extracoronal semi-precision attachments to enhance retention and stability, and the fabrication of complete dentures in cases of extensive tooth loss. Additionally, dental implants have emerged as a particularly relevant treatment modality, especially in highly complex cases where conventional prosthetic solutions may be insufficient. The choice of treatment plan is often tailored to the individual patient’s needs and preferences, considering factors such as the extent of tooth loss, the condition of the remaining teeth and supporting structures, and the patient’s overall oral health [9,10,11].

Although AHS is a common condition, there is a lack of agreement on its exact cause, and the literature often fails to provide a comprehensive analysis of its clinical manifestations. The current information available is limited to reporting that a diagnosis is tailored to eleven clinical features, covering only partial aspects of AHS. However, evidence suggests the potential for a more comprehensive, ontological, and multifactorial approach, extending beyond clinical aspects alone [12]. Rather than sparking controversy, this research aims to conduct a literature review of this information and analyze it, with the goal of proposing a conceptual model that defines AHS risk factors not solely by clinical features but also by exploring their causal relationships, benefiting both general practitioners and specialists, from the commonly termed “state of the art”.

## 2. Materials and Methods

### 2.1. Search Strategy 

The literature search strategy was implemented in several steps. Firstly, the databases PubMed, ScienceDirect, Ebsco, Google Scholar, Scielo, and Latindex were searched. Secondly, keywords, index terms, and free text terms were used in both English and Spanish, including “Kelly syndrome”, “Anterior hyperfunction”, “Combination syndrome”, “Treatment”, “Dentistry”, “Síndrome de Kelly”, “Hiperfunción anterior”, “Síndrome de combinación”, “Tratamiento”, and “Odontología”. Thirdly, the Boolean operators “OR” and “AND” were utilized to combine these search terms so as to broaden or narrow the search results. Various combinations of the search terms were applied, such as (“anterior hyperfunction syndrome” OR “anterior hyperfunction” OR “Kelly syndrome” OR “Combination syndrome”) AND “Treatment” AND “Dentistry”, and (“síndrome de Kelly” OR “hiperfunción anterior” OR “síndrome de combinación”) AND (“tratamiento” OR “odontología”). Lastly, the search was conducted on 27 January 2024 and included articles published from 1974 to 2024. 

### 2.2. Selection Criteria

The inclusion criteria encompassed clinical trials (case reports, case studies, retrospective studies); articles in English and Spanish; open-access articles and articles with full texts; and clinical and observational studies addressing aspects related to the assessment, diagnosis, and treatment of anterior hyperfunction in dentistry related to KS, AHS, and CS in dentistry as part of the intervention. Studies had to be published from 2000 to 2024 and available as the full text. Studies were evaluated based on methodological quality criteria, such as the study design, sample size, clarity in the description of the methods, and the validity of the results. 

The exclusion criteria encompassed preclinical research, in vitro studies, abstracts, theses, encyclopedias, book chapters, brief communications, opinion articles, and letters to the editor. We also excluded studies that were not directly related to diagnosis and those that did not report the diagnosis methods and treatment plan. 

## 3. Results

Given the limited number of substantive studies investigating the assessment and diagnosis modalities for AHS, a thorough analysis of the risk of bias is currently impractical, contrary to what was reported by Ogino et al. (2023) [13]. Therefore, a cutting-edge conceptual model is presented to enhance the comprehension and cultivate a deeper understanding of the subject. Additionally, case letters and literature and systematic reviews have been incorporated to further illustrate the conceptual model.

### 3.1. Assessment and Diagnosis of AHS Risk Factors

The analysis of risk factors for AHS throughout different studies shows an evolution in the understanding of the problem and in the recommendations for its management. In the initial studies, such as those by Kelly et al. (1972) and Saunders et al. (1979), an emphasis was placed on bone loss in the maxillary ridge and poor denture adaptation as key factors. These works focused on anatomical and mechanical aspects, highlighting problems such as papillary hyperplasia and the loss of the vertical dimension in occlusion [1,2].

Over time, the literature has expanded to include additional risk factors (Table 1). For example, Carlsson et al. (2004) and Buzayan et al. (2018) address psychosocial and economic aspects, suggesting that the management of the syndrome should consider not only the patient’s oral health but also their general well-being and socioeconomic status [14,15]. This trend towards a more holistic view is also reflected in studies such as those by Kumar et al. (2016), which point out the lack of an interdisciplinary approach and the importance of considering factors such as the intermaxillary distance and the need for additional implants to improve the prosthetic stability [11].

Recent studies, such as those by Akhtar et al. (2019) and Penitente et al. (2022), highlight the importance of long-term follow-up and the continuous adaptation of prostheses to prevent complications such as excessive bone resorption [43,45]. In addition, it is recognized that clinician-related factors, such as the quality of the diagnosis and treatment planning, are crucial for the successful management of the syndrome. Ogino et al. (2015, 2023) emphasize the need for adequate prosthetic planning, the use of keratinized tissue, and the implementation of an individualized treatment approach for each patient. The most recent literature advocates for a more integrated and multidimensional understanding of the problem, which includes anatomical, psychosocial, and economic considerations, as well as the importance of follow-up and continuous adaptation in patient care [10,13].

Most authors agree that establishing an accurate diagnosis is crucial in determining the appropriate treatment for AHS. However, they emphasize the importance of identifying all of the joint characteristics of a syndrome, determining hyperfunction from its collective features, although not primarily focusing on the diagnosis [10,23,24,30,42,45]. For example, Tolstunov (2007) suggested a classification based on Kennedy’s classification, focusing more on the treatment plan according to the type of edentulism present [3]. However, Saunders et al. (1979) and Langer et al. (1995) have pointed out that individual characteristics serve as a predictor. Moreover, the reviewed publications focus more on treatment rather than on diagnosis. There are even authors who, when diagnosing, refer to the premaxilla as a developmental condition. Although the term is widely used in dentistry and maxillofacial surgery to describe issues, the premaxilla as a distinct anatomical structure ceases to exist in terms of physical separation during the first year of life [19].

In addition to this, most authors describing the diagnosis do so through an initial treatment plan that starts with mounted study models, panoramic radiographic films, cephalometric radiographic films, and intraoral photographs. The mandibular model is duplicated, and a diagnostic wax-up is performed. Furthermore, Madan et al. (2006) agree that patients with edentulous maxillae and a partially edentulous mandible are at higher risk [21]. They underscore the significance of examining both medical and dental records, performing a comprehensive clinical and radiographic assessment of the hard and soft tissue related to prosthesis use, addressing any existing inflammation, and evaluating the patient’s susceptibility to dental caries, their periodontal condition, and their oral hygiene practices [27,28].

On the other hand, Magureanu et al. (2009) state that occlusal pressure begins at the occlusal level through direct contact between the teeth or between the tooth, food, and tooth. Under normal and physiological conditions, during mastication, the pressure is initially absorbed by the lateral crest, which is capable of withstanding vertical pressure. However, the pressure exerted in the frontal region that becomes horizontal is not functional and turns out to be destructive for the support structures. This explains the considerable resorption observed in the anterior area of the maxilla, which acquires a fibrous appearance, typical of anterior hyperfunction syndrome [25].

It is crucial to evaluate the time for which a patient has been without prostheses and to examine the temporomandibular joint (TMJ), looking for changes in the condyle of the lower jaw in relation to the glenoid cavity. The extracted teeth and their impact due to pathological migration should be considered. Occlusal analyses and habit detection are included to identify alignments or irregularities and bite repositioning patterns. Tools like articulating paper can highlight premature contact points, which can lead to stress and inflammation states in the periodontal ligament and non-carious lesions such as abfractions, attritions, and dental fractures, reducing the height of the interdental bone crests. However, this method may not always reveal underlying pathologies, so the detection of occlusal pathologies is often carried out through the clinical history and TMJ examinations, such as bruxism or clenching. Devices like electromyography (EMG) are also suggested to measure muscle activity, helping to identify the root cause of symptoms, although its limitation lies in its dependence on subjective patient reports. Alternatively, imaging modalities like orthopantomography, periapical X-rays, magnetic resonance imaging (MRI), or computed tomography (CT) scans provide comprehensive insights into both hard and soft tissue. Incorporating cephalometric analysis helps to identify abnormal growth or displacement. The use of study models and semi-adjustable articulators allows for a detailed, although not precise, evaluation of movement patterns, offering a three-dimensional perspective on the jaw’s position relative to the skull base—crucial for treatment planning. Additionally, diagnostic wax-ups with a prosthodontic approach facilitate the assessment of interocclusal space discrepancies, predicting the necessity for pre-prosthetic surgery. Furthermore, interdisciplinary meetings for case evaluation, considering the diverse characteristics of each patient, are highly recommended. It is insufficient to assess a patient solely by examining individual aspects such as the teeth, resins, and crowns, without conducting a comprehensive overall assessment [32].

This aids in determining whether treatments will be additive or subtractive, contingent on the extrusion of the lower teeth. Photographs not only document the patient’s initial state but also, when appropriately calibrated, serve as a tool for standard measurements from digital designs. This follows the same principle as in digital dentistry, albeit through a manual approach.

### 3.2. Epidemiology

According to Kelly’s reports, this problem directly affected 26% of the patients, accounting for a quarter of the consultations in the prosthodontics clinic. This was also confirmed by Shen et al. (1987) and Gonçalves et al. (2007), who reported that, of the total patients reviewed, the frequency of AHS characteristics was 25%. Moreover, the presence or absence of prostheses was not determinant regarding the incidence of pathological changes [17,22].

On the other hand, Kilicarslan et al. (2012) reported a prevalence of 7–9%, although the most relevant aspect was that maxillary changes have a strong correlation with AHS and should be considered significant, as even the type of prosthetic occlusion significantly influences the loss of mandibular and maxillary alveolar bone. Furthermore, bone loss in the anterior maxillary area is the most important sign [29].

However, despite the previously reported data, Bagga et al. (2019), in a cross-sectional observational study evaluating 99 patients, suggest that AHS either does not exist or is rare. Nevertheless, the results show selection and interpretation biases, and it is inferred that the clinical characteristics are due to other causes, resulting in the extrapolation of the results. This also establishes a basis for considering that the reported etiology of AHS is multifactorial [44].

Palqmvist et al. (2003) also point out that Dorland’s Illustrated Medical Dictionary does not list “combination syndrome” among the numerous syndromes that it describes, indicating that such theories might be speculative due to their multifactorial etiologies. While the individual characteristics linked to “combination syndrome” are recognized, the specific extent or combinations in which they manifest remain undefined [20].

### 3.3. Hyperfunction Oral State: A Comprehensive Overview for a Conceptual Model

**Figure 4:**
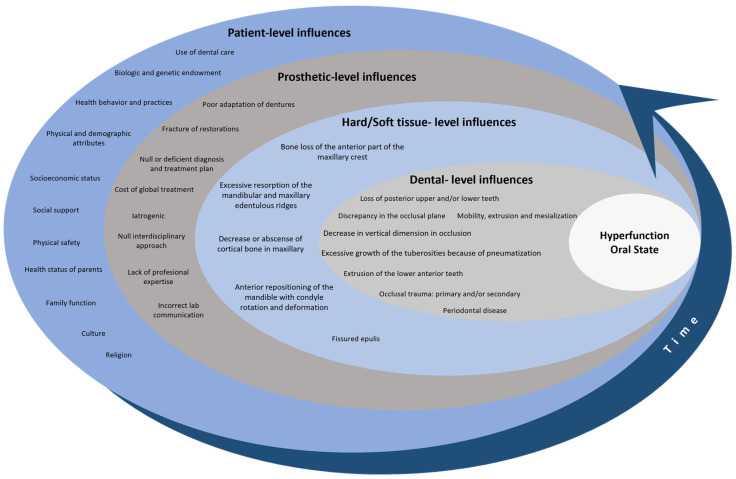
Conceptual model of hyperfunction oral state risk factors. The image depicts a conceptual model that represents the accumulation of influences over time at the patient level, prosthetic-level, tissue level, and dental level via a complex interplay between patient-related factors, the mechanics of their prosthetics, and the health of their hard and soft tissue, all of which are influenced by the individual’s occlusion. Source: the authors.

The existing literature on the relationship between risk factors and anterior hyperfunction syndrome was comprehensively reviewed. Our analysis extends beyond a mere examination of the published works, delving into a profound exploration of the clinical findings and experience. In this investigation, we do not view the characteristics of AHS as mere coincidences or happenstance and associated with a syndrome according to Bagga and Plaqmvist. The literature overview instead indicates that the syndrome is a hyperfunction oral state *(*HOS*)*. The word “hyperfunction” originates from two elements with roots in Ancient Greek and Latin. The prefix “hyper-” comes from the Greek “ὑπέρ”, meaning “over”, “above”, or “beyond”, and is commonly used in English to denote something that exceeds normal limits or is excessive in nature. The second part of the word, “function”, comes from the Latin “functio”, which in turn is based on the verb “fungi”, meaning “to perform” or “to accomplish”. Together, “hyperfunction” is literally translated as a function or activity that is excessive or goes beyond what is normal and is applied in contexts where there is increased or excessive activity compared to typical standards [46,47]. The HOS in patients results from a cascade of events that occur simultaneously in the oral system across four levels and do not necessarily follow a chronological order. These levels include aspects related to the patients and rehabilitation, as well as hard and soft tissue and occlusion. The conventional clinical aspects proposed by Kelly, Saunders, and Tolstunov are integrated within these multifaceted levels (Figure 4) and based on Fisher–Owens’ conceptual model for oral health [48].

#### 3.3.1. Patient-Level Influences

The patient level considers the examination of risk factors extending beyond the conventional analysis of the literature to encompass a thorough exploration of clinical findings based on practical experience [49]. It encompasses a spectrum ranging from patient comorbidities, habits, and genetic and epigenetic aspects to familial, social, and demographic influences. Timely patient attention is crucial, considering issues such as limited access to quality dental services, resulting in a lack of preventive care [50,51]. Cultural and environmental factors may lead individuals with access to dental services to seek treatment only when experiencing visible issues or pain. Unfortunately, delayed visits often result in extractions, leading to complex prosthetic interventions and contributing to anterior hyperfunction pathologies [52]. Elevated treatment costs and time constraints further discourage patient commitment. Inadequate oral hygiene exacerbates diseases affecting both hard and soft support tissue. The extraction of posterior teeth destabilizes the occlusion, creating a dental cascade effect akin to a domino effect, as previously described [53].

#### 3.3.2. Prosthetic-Level Influences

**Figure 5:**
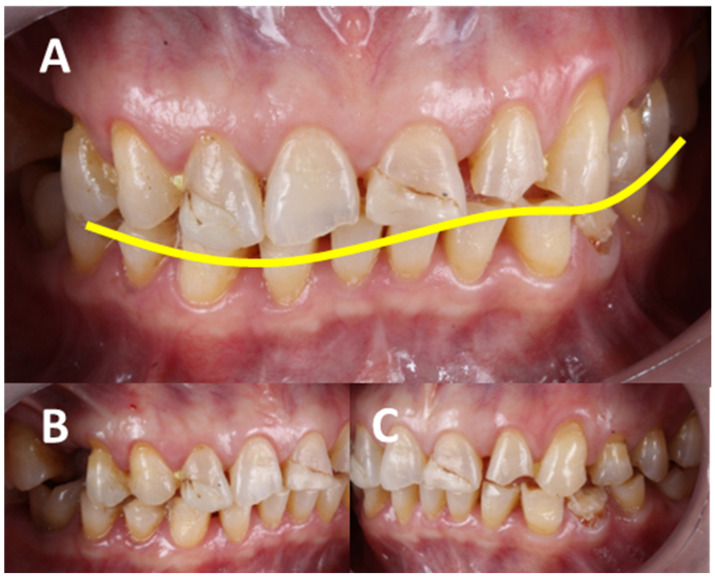
**Intraoral photographs depict the restorative ‘death spiral’**. They illustrate the progression towards dental extraction. Understanding the factors involved in the dental re-restoration cycle is crucial due to its implications in terms of increasing the size of restorations and the variability in diagnostic accuracy and treatment planning among professionals, as well as the biological and structural impacts on teeth and the rising costs of dental care. This is essential in determining patient prognosis and an early hyperfunction state. (**A**) shows defective restorations related to dental attrition, fractured resins, high biofilm accumulation, abfractions, and an inverted smile arc. (**B**,**C**) show the absence of posterior teeth in the first sextant. Source: the authors.

At the prosthetic level, adding to the patient influences, the role of inadequately trained dental professionals becomes paramount in shaping the trajectory of anterior hyperfunction. A lack of clinical judgment, unnecessary treatments, insufficient interdisciplinary approaches, and inadequacies in clinical and laboratory equipment contribute significantly to this issue. Poorly executed treatments such as ill-fitted crowns, endodontic deficiencies, and improper material usage in high-stress areas, disregarding biological and mechanical principles, further complicate the scenario [54]. The tendency to address periodontal pathologies with simple prophylaxis, without consulting periodontists, often leads to the gradual deterioration of the masticatory complex, both in the soft and hard tissue, causing occlusal disorders. These disorders, in turn, aggravate anomalies in the hard and soft tissue, forming a cyclical pattern termed the “restorative death spiral”, indicating the journey that an intervened tooth undergoes until extraction (Figure 5) [55].

**Figure 6:**
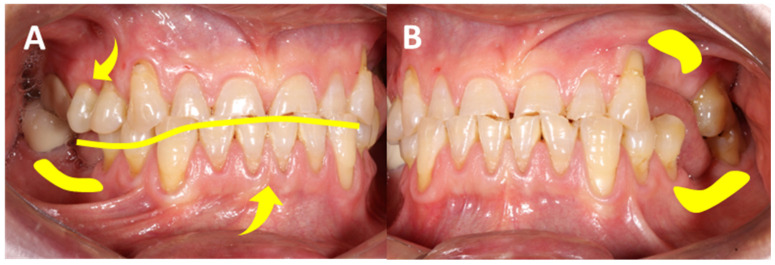
**Prevalent clinical characteristics in patients before the establishment of HOS**. (**A**,**B**) Lateral observations, both right and left, reveal discrepancies in the occlusal plane originating from the anterior teeth in an edge-to-edge bite position, highlighting the reduction in vertical dimension caused by these teeth. This situation is identified by the authors as an occlusal pathology, including ACF and an edge-to-edge bite. These conditions predispose patients to attrition and abfraction, which can lead to dental fractures, bone loss, and a significant reduction in marginal gingiva, predominantly exposing the mucosa. Additionally, the absence of posterior teeth increases the stress in the anterior region, and their non-replacement leads to the pathological migration of the teeth and the loss of alveolar height. Source: The authors.

On the other hand, the diversity of the degrees of bone resorption may be related not only to the poor fitting of prostheses or inadequate manufacturing but also to metabolic changes, hormonal factors, nutritional factors, or traumatic factors, among others [56]. Therefore, in agreement with Bagga et al., it is prudent to consider that damage to the hard and soft tissue cannot be limited to just one factor [45]. However, it is very risky to deny the clinical evidence that shows the effect of prostheses on the alveolar ridge. This implies that an early diagnosis of trends in the oral status, which indicate a possible risk of suffering from the marked expression of HOS, could and should be made. Therefore, it is asserted that it is unnecessary to wait for a tooth to be extracted to undertake an interdisciplinary approach. Instead, based on clinical signs, it is possible to obtain a timely diagnosis and, consequently, offer a better personalized treatment plan (Figure 6).

#### 3.3.3. Hard/Soft Tissue-Level Influences

**Figure 7:**
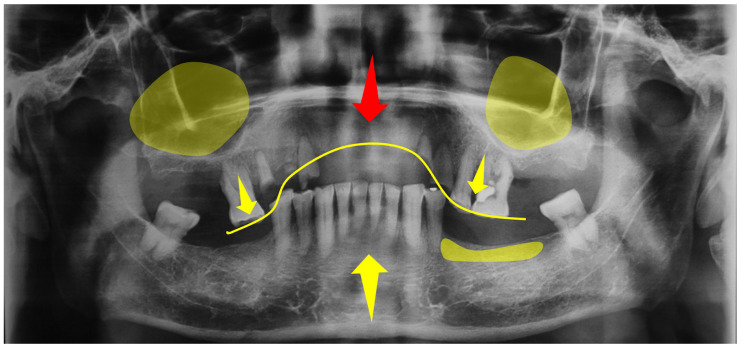
**Orthopantomography that highlights areas potentially signaling bone loss**. Yellow arrows are used to pinpoint specific instances of tooth extrusion. The red arrow points to the maxillary area, which represents bone loss and atrophy. Additionally, a yellow line traces the curve of Spee, highlighting the natural curvature of the biting surfaces of the teeth and providing insight into the alignment and bite relationship between the upper and lower dental arches. The X-ray image is critical for diagnostics for dental professionals in assessing the overall oral health of a patient, aiding in treatment planning, and monitoring dental and skeletal development. Source: the authors.

After tooth loss, the effects on both hard and soft tissue become crucial for the development of anterior hyperfunction. Tooth absence, regardless of its location, leads to alveolar ridge atrophy. In the posterior maxillary region, the lack of bone stimulation due to the absence of the periodontal ligament results in bone resorption and, specifically in the posterior maxilla, subsequent maxillary sinus pneumatization. Although the growth of tuberosities has traditionally been attributed to removable prostheses, due to cohesive retention forces and the perpendicular effect in the fulcrum area, conclusive evidence for this cause-and-effect relationship is still lacking. However, the atrophy associated with tooth absence and pneumatization leaves only the basal bone, complicating the adaptation of future prostheses and surgical implant treatments due to insufficient bone for primary stability. The maxillary tuberosity has been identified as the main site of maxillary growth. The increase in tuberosity—along with tooth loss, which directly correlates with bone loss and, when combined with unstable prostheses, accelerates atrophy—makes excessive tuberosity growth a distinctive feature [57] (Figure 7). Furthermore, a reduction in the anterior process of the maxilla has been observed with age. On the other hand, the mandible, being more cortical and denser than the maxilla, better withstands the impact of mastication. An adequate number of roots in the maxilla to withstand constant contact with the mandibular teeth is crucial. Despite this, the mandibular teeth, prone to mobility, fractures, or root failures due to their smaller size, show patterns of extrusion of the anteroinferior teeth, phenomena correlated with HOS and AFC, as well as with occlusal trauma and occlusal plane changes due to mandibular rotation.

#### 3.3.4. Dental-Level Influences

**Figure 8:**
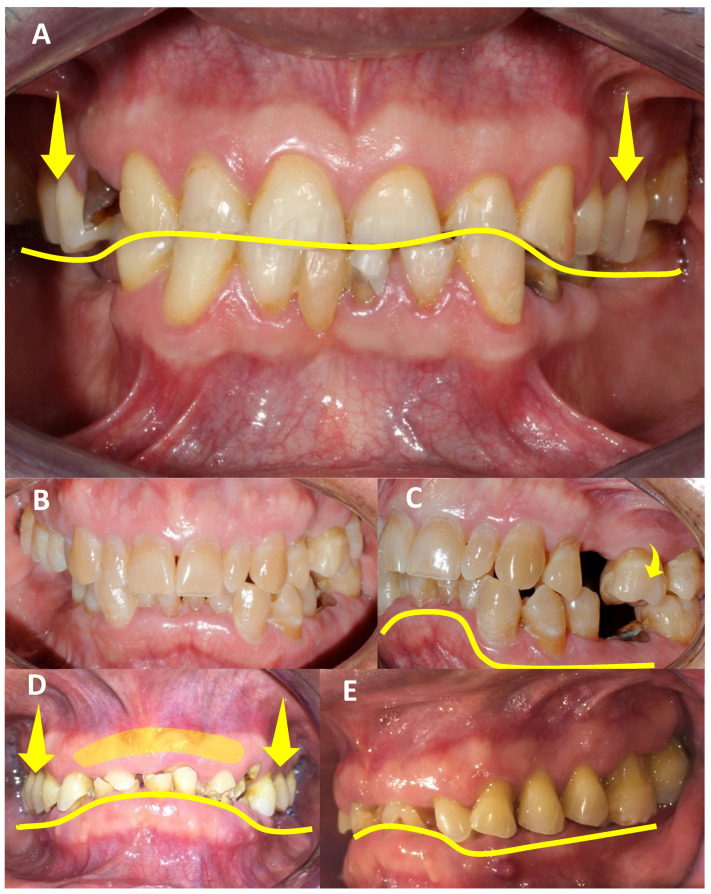
**Intraoral photographs show how occlusion is altered due to tooth absence**. (**A**) shows an increased Spee’s curve, with the extrusion of the upper posterior teeth due to the absence of opposing teeth and signs of attrition. (**B**,**C**) display crowding in the fifth sextant with a pronounced increase in Spee’s curve, the absence of posterior teeth with the extrusion of the opposing teeth, as well as residual dental roots and signs of gingival recession with the exposure of the dentin necks. (**D**,**E**) reveal the severe destruction of the anterior teeth, complete absence of the lower posterior teeth, and extrusion of the upper opposing teeth, with an increase in tuberosities. Additionally, it shows residual dental roots, multiple fistulas due to secondary caries, and severe attrition. Source: the authors.

Finally, at the dental level, patients may lose not just a single tooth but several. If the loss occurs in the posterior region, the adjacent teeth are prone to movement due to anterior force components, compounded by proprioceptive factors, leading to the extrusion of antagonists. When teeth are lost prematurely and not adequately restored, this becomes essential. Stress emerges as a significant contributing factor to HOS. The aspects of life that typically bring joy and tranquility, such as work, studies, or family, are transformed into weapons that are used against us. Stress disrupts our state of well-being, creating a profound imbalance in the central nervous system, closely linked to occlusal pathologies. The tendency of dentists to underestimate teeth clenching or grinding is noteworthy, overlooking these as primary causes of tooth loss today. Previously dominated by caries, the combination of occlusal and periodontal pathologies leading to secondary occlusal trauma results in the premature fatigue of the supporting tissue. Consequently, we observe a rising trend of extracting otherwise healthy teeth, free from caries, due to support loss or periapical abscesses from periodontal diseases. The interconnected factors—periodontal disease, occlusal pathology, patient neglect, inadequate clinical or interdisciplinary management, and environmental factors—culminate in tooth loss, directly associated with the HOS. This raises the subsequent question: what comes after tooth extraction? The logical progression involves replacement to counteract movements induced by AFC and the proprioceptive responses of the supporting tissue. This helps to prevent the tooth from extruding or migrating in search of essential contact and stimulation. In fact, the equation described emerges as a significant risk factor in published articles linked to the HOS (Figure 8).

## 4. Discussion

The current state of the art in dentistry for AHS involves considering the theoretical and clinical evidence presented in this review, which suggests a hyperfunction oral state with the clinical features previously reported. This is relevant because a preventive approach can be applied in diagnosis based on the individual clinical condition of each patient before the AHS features are established per se [58].

This means that the conceptual model of HOS can help general practitioners and specialists to understand the progression of certain characteristics, even before they appear. In other words, dentists should not wait for more severe clinical manifestations to anticipate their possible evolution in the oral deterioration of patients [59]. However, numerous tools are at the disposal of all dentists, and, while some may incur higher costs, their value lies in facilitating precise evaluation, diagnosis, and treatment planning. Clinical findings stand out as the paramount tool for dentists, enabling the identification of the exact cause of symptoms—an imperative for effective treatment. An inaccurate diagnosis, disregarding the risk factors, can lead to ineffective treatment or exacerbate the situation. Emphasizing the adage that “the eyes see what the mind knows”, continuous education in diagnosis and occlusion is vital for the accurate treatment of any oral disease according to the AHS features.

Dentists are strongly encouraged to actively engage in courses and seminars focused on these areas, thereby enhancing their proficiency in handling complex cases. The objective is not to gain knowledge at the patient’s expense but to apply sound expertise in each case. While experience holds significant value, in its absence or in conjunction with clinical judgment, it is advisable to refer patients to specialists to ensure the provision of optimal care.

**Figure 9:**
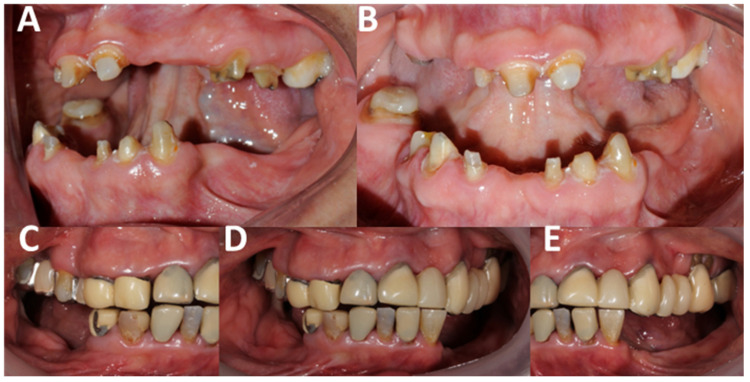
**Impact of inadequate diagnosis and planning on fixed prosthesis treatment: a focus on temporization**. The direct effect on the remaining teeth and both the hard and soft tissue is evident because of the low quality of diagnosis and planning. (**A**,**B**) A lack of temporization with exposed abutments, showing poor occlusal stability. They also present bone resorption at the mandibular ridge, in addition to a state of anterior hyperfunction with the extrusion of the lower anterior teeth, as well as anterosuperior mandibular rotation. (**C**) displays multiple metal–ceramic restorations with porcelain chipping, marginal leakage, substrate color change, and metal exposure. (**D**,**E**) show a 6-unit fixed partial denture with the root exposure of the abutment at 2.6. Additionally, it shows the collapse of the mandibular alveolar crest and aberrant frenulum. Source: the authors.

Preventive treatments embody characteristics such as promptness, swiftness, safety, conscientiousness, and timeliness. Any prosthetic treatment adheres to a structured process encompassing three distinct phases, each comprising five essential aspects. The initial phase is the pre-intervention stage, encompassing tasks such as diagnosis, consultation/referral, effective communication with the patient, meticulous treatment planning, and obtaining informed consent. The second phase is identified as the intervention level and involves specific actions: the preparation of the treatment area, precision in taking impressions, temporization, seamless communication with the dental technician, and the finalization of prosthetic dental restorations. The concluding phase is the post-intervention stage. At this juncture, it involves providing thorough instructions for oral and dental prosthesis care, scheduling follow-up appointments, maintaining a comprehensive dental record, and upholding the confidentiality of the patient’s treatment record. In the field of dental and prosthetic treatment, dentists are obligated to adhere to strict diagnostic, ethical, and legal guidelines to avoid malpractice in all of the previously described phases. However, it is necessary to focus on diagnosis with the aim of achieving the correct treatment plan to ultimately achieve success and patient satisfaction (Figure 9) [26].

## 5. Conclusions

AHS is commonly associated with specific characteristics. However, the HOS more specifically represents the gradual deterioration of the stomatognathic system, identified by clinical signs, which may initially appear in isolation but will eventually have a significant impact on the patient’s quality of life. Prevention and early diagnosis are essential, as they are the most valuable tools for the dentist. For this reason, it is imperative to reach a consensus on the risk factors and their cause-and-effect relationships. The conceptual model proposes an in-depth approach that is open to continuous improvement. This will allow for timely intervention and help to establish a predictable treatment plan.

## Figures and Tables

**Table 1:** Risk factors for AHS. They show evolution in its understanding and management. The factors include anatomical, mechanical, psychosocial, and economic aspects. The need for a multidisciplinary approach, follow-up, and personalized adaptation is highlighted.

Authors	AHS Risk Factors
Kelly, 1972 [1]	Bone loss of the anterior part of the maxillary crest; excessive growth of tuberosities; papillary hyperplasia in the hard palate; extrusion of the lower anterior teeth; excessive resorption of the mandibular edentulous ridges; excessive force; shearing forces; inadequate denture base coverage; underlying systemic causes
Saunders et al., 1979 [2]	Loss of vertical dimension in occlusion; discrepancy in the occlusal plane; anterior repositioning of the mandible; poor adaptation of prostheses; epulis fissuratum; periodontal disease; systemic disease; caries; oral hygiene
Schmitt et al., 1983 [16]	Occlusal stress
Shen et al., 1989 [17]	Periodontal disease
Langer et al., 1995 [5]	Poorly designed mandibular RPD
Jameson et al., 2001 [18]	Excessive anterior force
Cabianca et al., 2003 [19]	Posterior tooth loss; unstable occlusal plane
Palmqvist et al., 2003 [20]	Supraerupted maxillary molars; artificial denture teeth;
Carlsson et al., 2004 [14]	Anatomy; psychosocial aspects, mechanical devices; gender; age; facial morphology; duration of edentulousness; denture wearing habits; number of dentures worn; oral hygiene; parafunctions; occlusal loading; denture quality; nutrition; general health; medication; systemic; diseases; osteoporosis; corticosteroid treatment for asthma
Madan et al., 2006 [21]	Combination of complete maxillary dentures opposing class I mandibular RPD; retaining weak posterior teeth as abutments; conventional lower denture
Gonzalves et al., 2007 [22]	Lack of prosthesis adaptation
Tolstunov, 2007 [3]	Type of edentulism
Flanagan et al., 2008 [23]	Inappropriate treatment
Daher et al., 2008 [24]	Lack of professional expertise
Magureanu et al., 2009 [25]	Excessive pression in frontal region
Tolstunov, 2009; 2011 [6,9]	Abnormal traumatic occlusal forces
Gerritsen et al., 2010 [26]	Low quality of life
Jyoti et al., 2010 [27]	Null or deficient diagnosis and treatment plan
Rao et al., 2011 [28]	Deficient mandibular RPD
Kilicarslan et al., 2012 [29]	Edentulous maxilla
Peñarrocha et al., 2012 [30]	Marginal bone loss related to maxillary atrophy class
Feng et al., 2012 [31]	Edentulous maxilla opposed to natural mandibular anterior teeth; distal-extension RPD
Ibrahim et al., 2013 [32]	Low quality of bone in edentulous maxilla; deficient diagnosis; time
Resende et al., 2014 [33]	Mandibular RPD with inadequate technical quality; RPD absence
Carlino et al., 2014 [34]	Complete maxillary denture opposing complete denture attached to implants by bars or ball attachments; biomechanical stress to anterior maxilla of implants supported by prosthesis; lack of pre-prosthetic surgical intervention; no consideration of occlusion, vertical dimension, or occlusal plane; lack of follow-up
Barroeta et al., 2015 [35]	Deficient diagnosis; inadequate oral rehabilitation, lack of professional expertise; absence of lower RPD, inadequate lower RPD; lack of preventive treatment; decreased vertical dimension; maladaptation of the upper prosthesis; inverted prosthetic plane
Oliveira et al., 2015 [36]	Lack of diagnosis of the patient’s characteristics before treatment; combination of an upper tissue-supported prosthesis with lower RPD; inadequate occlusal schemes in prostheses; failure to eliminate the contact between the anterior teeth and the lower teeth
Narwal et al., 2015 [37]	Increasing pressure on the premaxillary alveolar ridge; loss of adequate posterior occlusal
Patel et al., 2015 [8]	Incorrect and inappropriate occlusal diagnosis for treatment planning
Rajendran et al., 2015 [38]	Lack of evaluation of dental history and the condition of the remaining mandibular anterior teeth; stress on the maxillary ridge, angle class III jaw relationships, parafunctional habits, and prolonged function with mandibular anterior teeth; degenerative changes in edentulous regions; inadequate treatment planning; failure to maintain oral tissue health; insufficient diagnosis, planning, and treatment implementation
Ogino et al., 2015 [10]	Patients failing to attend follow-up appointments; inadequate relationship between implant position and optimal artificial tooth positions; low quality and quantity of bone; absence of keratinized tissue; non-personalized treatments
Reddy et al., 2016 [39]	Lack of maxillary denture adaptation; need for replacement of maxillary denture; lack of mandibular denture adaptation; sex
Kumar et al., 2017 [11]	Inter-arch distance and relationship; lack of or null analysis of the anatomy of the maxilla using all tools available, including diagnostic models, X-ray images (radiographs, CBCT); incorrect impression technique; financial limitations for additional implants; lack of bone to support an adequate number of implants; loss of supporting structures for the lips and surrounding tissue; avoidance of bone grafting; no use of a tissue implant-supported hybrid denture as a less expensive and simpler option, within certain guidelines
Stevkovska et al., 2017 [40]	Lack of interdisciplinary therapy approach; delayed diagnosis; deficient treatment plan
Sharma et al., 2018 [41]	Lack of maxillary denture adaptation; not replacing the maxillary denture
Buzayan et al., 2018 [15]	Presence of a large torus palatinus and enlarged tuberosities; partially dentate mandibular arch with remaining anterior teeth; compromised sulcus depth: lack of pre-prosthetic surgical procedures; economic factors affecting treatment options; patient’s desires influencing the treatment plan; bone availability for dental implants; patient’s general health considerations; potential for progressive destructive changes in oral tissue without proper management
Verma et al., 2018 [42]	Reduced posterior occlusal contact; lack of use of implant-retained prostheses in the mandibular posterior area; extraction of upper posterior teeth; imbalanced occlusion
Akhtar et al., 2019 [43]	Inadequate surgical and prosthodontic treatment and lack of follow-up; tooth extrusion associated with RPD wearing; unsatisfactory lower dentures; non-simultaneous rehabilitation of residual arches; presence of preexisting signs before the provision of removable dentures; alveolar bone resorption as a natural phenomenon post-tooth loss; lack of scheduled follow-up sessions and proper guidance on denture care; poor preservation of posterior occlusion; inadequate treatment modalities, including poor-quality RPDs; failure to address papillary hyperplasia; lack of surgical procedures and proper impression techniques for flaccid tissue; poor fit, hygiene, and occlusion maintenance; insufficient use of implants to convert mandibular Kennedy Class 1 and 2 to Class 3, which could improve the masticatory efficiency, stability, and aesthetics; lack of implant-supported RPDs to reduce bone resorption
Bagga et al., 2019 [44]	Unsatisfactory dentures; periodontitis; maxillary complete dentures opposing mandibular anterior teeth
Penitente et al., 2022 [45]	Excessive bone resorption in the maxilla; occlusal architecture rearrangement; discrepancies between dental arches; divergent bone quality between maxilla and mandible; faster bone loss post-tooth extraction; greater bone loss with complete dentures; insufficient implant use; inadequate prosthetic and surgical planning; imbalanced occlusion; inadequate prosthesis material; insufficient posterior stabilization of the mandible
Ogino et al., 2023 [13]	Shift in mastication to anterior regions; excessive anterior occlusal function; occlusal trauma; lack of timely implant treatment; traumatic occlusion by preserved anterior teeth; excessive bone resorption in maxilla; lack of posterior occlusion; insufficient follow-up care; inadequate prosthetic treatment; loss of posterior occlusion support; improper implant placement; lack of cross-arch stabilization; inadequate bone quality and quantity; poor oral hygiene control; absence of keratinized tissue; inadequate prosthetic design; lack of individualized treatment approach

## Data Availability

Not applicable.
